# Bioinformatics Analysis Highlights Five Differentially Expressed Genes as Prognostic Biomarkers of Cervical Cancer and Novel Option for Anticancer Treatment

**DOI:** 10.3389/fcimb.2022.926348

**Published:** 2022-06-17

**Authors:** Hongtu Cui, Ruilin Ma, Tao Hu, Gary Guishan Xiao, Chengjun Wu

**Affiliations:** ^1^School of Biomedical Engineering, Dalian University of Technology, Dalian, China; ^2^School of Pharmaceutical Science and Technology, Dalian University of Technology, Dalian, China

**Keywords:** bioinformatics analysis, cervical cancer, DEGs, prognosis, anticancer drug

## Abstract

Cervical cancer is one of the most common gynecological malignancies and is related to human papillomavirus (HPV) infection, especially high-risk type HPV16 and HPV18. Aberrantly expressed genes are involved in the development of cervical cancer, which set a genetic basis for patient prognosis. In this study, we identified a set of aberrantly expressed key genes from The Cancer Genome Atlas (TCGA) database, which could be used to accurately predict the survival rate of patients with cervical squamous cell carcinoma (CESC). A total of 3,570 genes that are differentially expressed between normal and cancerous samples were analyzed by the algorithm of weighted gene co-expression network analysis (WGCNA): 1,606 differentially expressed genes (DEGs) were upregulated, while 1,964 DEGs were downregulated. Analysis of these DEGs divided them into 7 modules including 76 hub genes. Kyoto Encyclopedia of Genes and Genomes (KEGG) and Gene Ontology (GO) enrichment analysis revealed a significant increase of genes related to cell cycle, DNA replication, p53 signaling pathway, cGMP-PKG signaling pathway, and Fanconi anemia (FA) pathway in CESC. These biological activities are previously reported to associate with cervical cancer or/and HPV infection. Finally, we highlighted 5 key genes (*EMEMP2, GIMAP4, DYNC2I2, FGF13-AS1*, and *GIMAP1*) as robust prognostic markers to predict patient’s survival rate (p = 3.706e-05) through univariate and multivariate regression analyses. Thus, our study provides a novel option to set up several biomarkers for cervical cancer prognosis and anticancer drug targets.

## Introduction

Cervical cancer is one of the most common gynecological malignancies (Small et al., 2017), of which cervical squamous cell carcinoma (CESC) accounts for more than 80% of total cases (Cheng et al., 2020). Cervical cancer has become one of the most susceptible and fatal cancers in women, mainly through sexual contact (Ojesina et al., 2014; Szymonowicz and Chen, 2020). According to the latest data from the International Cancer Center, about 600,000 new cases of cervical cancer have been reported worldwide in 2020; the reported death cases were more than 340,000 (Sung et al., 2021). Earlier studies have shown that the occurrence of cervical cancer is directly related to the persistent infection of high-risk human papillomavirus (HPV) (Schiffman et al., 2016); more than 99.7% of cervical cancer patients were infected by high-risk HPVs. High-risk subtype HPV16 and HPV18 are the most prevalent types (Walboomers et al., 1999). High-risk HPVs generate E6 and E7 oncoproteins. The E6 protein binds to the cellular factor p53, causing p53 degradation, thereby disrupting cellular apoptosis. The E7 protein interacts with pRB, causing pRB inactivation and altering the cell cycle regulatory pathways. Together, the E6 and E7 oncoproteins cause the transformation of infected cells and finally accumulate mutations to further develop into cancer cells (Burd, 2003).

As the most efficient screening methods Pap test and HPV test are widely used for cervical cancer diagnosis in the clinic (Kessler, 2017), most of the patients can be diagnosed and treated at an early stage of cervical cancer progression. Unfortunately, there is no efficient treatment for advanced or recurrent cervical cancer (Wang et al., 2014; Yang et al., 2020). To date, the common treatment of cervical cancer includes surgical resection, radiotherapy, and chemotherapy (Ellenson and Wu, 2004; Hazell et al., 2018), but the prognosis of patients does not meet expectations. Especially, the 5-year survival rate of patients is even less than 10% (Eskander and Tewari, 2014). Therefore, it is important to identify novel anticancer drug targets to improve therapy efficiency. Moreover, novel prognostic biomarkers are needed to promote the survival rate of cervical cancer patients.

Recent studies focus on screening differentially expressed genes (DEGs) but ignore the complex networks among genes and the gene-related clinical phenotypes (Liu et al., 2019). However, more and more evidence suggests that the arising of cervical cancer involves multiple abnormally expressed genes (Zhou and Wang, 2015; Bahrami et al., 2018). The high-throughput data mining algorithm weighted gene co-expression network analysis (WGCNA) identifies biological key modules by using high-throughput gene expression data (Langfelder and Horvath, 2008). In recent years, WGCNA has been increasingly used in the research of tumor markers. For example, Xing et al. (2020) used WGCNA to find PHY906 and CPT11 as key genes for colon cancer.

In this study, we obtained the DEG expression profiles from the public database The Cancer Genome Atlas (TCGA) to construct a co-expression network to identify cervical cancer progression-related hub genes. These highlighted hub genes can be applied to predict the 3- and/or 5-year survival rates of cervical cancer patients. The potential role of these genes as biomarkers needs to be further studied, and it may also provide a theoretical basis for the prognosis assessment of cervical cancer patients.

## Materials and Methods

### The Cancer Genome Atlas Data Preprocessing

The plan of the study is shown in **Figure S1**. The original cervical cancer expression data (Htseq-counts), standardized data (Htseq-FPKM), and clinical data were obtained from TCGA database (https://cancergenome.nih.gov/), including three normal cervix tissue samples and 304 cervix cancer tissue samples. The clinical data contain information of phenotypes, including the age (20–88 years), gender (men, women), clinical stage (stage IV, stage III, stage II, stage I), neoplasm histologic grade (G4, G3, G2, G1, GX), survival time (0–6,408 days), body mass index (13–70), HPV status (positive, negative), smoking history grade (1–3), and vital status (alive, dead). Low-expression samples in raw data were excluded using the filterByExpr in the edgeR package (Law et al., 2016).

### Analysis of Differentially Expressed Genes

The edgeR package, DESeq2 package, and limma package are used to analyze gene expression differences between normal cervical tissue samples and cancer samples (Robinson et al., 2010; Anders and Huber, 2010; Law et al., 2014). The analyzed genes with threshold p value <0.05 and |log2fold change (FC)| >1 were selected as the final DEGs of cervical cancer.

### Gene Function Enrichment Analysis

Gene function enrichment analysis is adopted to compare genes or genomes with functional databases for overexpression analysis and functional annotation. GO database is mainly for the study of cellular component (CC), molecular function (MF), and biological process (BP) (The Gene Ontology Consortium, 2019). KEGG database is for understanding the functions and applications of biological systems according to genomics or the information at the molecular level (Kanehisa et al., 2017). In this study, the enrichGO and enrichKEGG functions in software R package cluster profile (Yu et al., 2012) were used to conduct enrichment analysis and pathway analysis of the DEGs to identify the BP of significant GO (p < 0.05) and the significant KEGG pathways (p < 0.05).

### Weighted Gene Co-Expression Network Analysis

The WGCNA package in R software was selected to conduct the process of WGCNA (Langfelder and Horvath, 2008). The construction steps of the network mainly include the gene co-expression similarity matrix calculation, the adjacency function of the gene network calculation, the soft threshold selection, the topological overlap matrix (TOM), the dissimilarity matrix calculation, the gene module by dynamic branch cut method calculation, and the correlation analysis between the gene module and external information.

### Core Gene Screening

We use Cytoscape software to visualize the gene network in the module (Doncheva et al., 2019); the highest connectivity genes are identified as hub genes according to the connectivity between genes in the module.

### Survival Analysis

The survival package of R software is used for survival analysis of hub genes. According to the median value of the gene expression level, cervical cancer samples were divided into two groups. The survival curves of each group were generated.

### Construction of Prognostic Markers of Cervical Cancer Based on Key Genes

Univariate Cox proportional hazards regression analysis was adopted to assess the relationship between hub genes and survival rate. The estimation of the prognostic risk score of each cervical cancer patient was analyzed by multivariate Cox regression analysis. The risk score model was built using the coxph function of the R software survival package. According to the risk score, patients can be divided into low-risk and high-risk groups. The software R survival package generated the survival curves of the two groups.

## Results

### Differential Analysis Selected 1,606 Upregulated Genes and 1,964 Downregulated Differentially Expressed Genes

**Figure 1:**
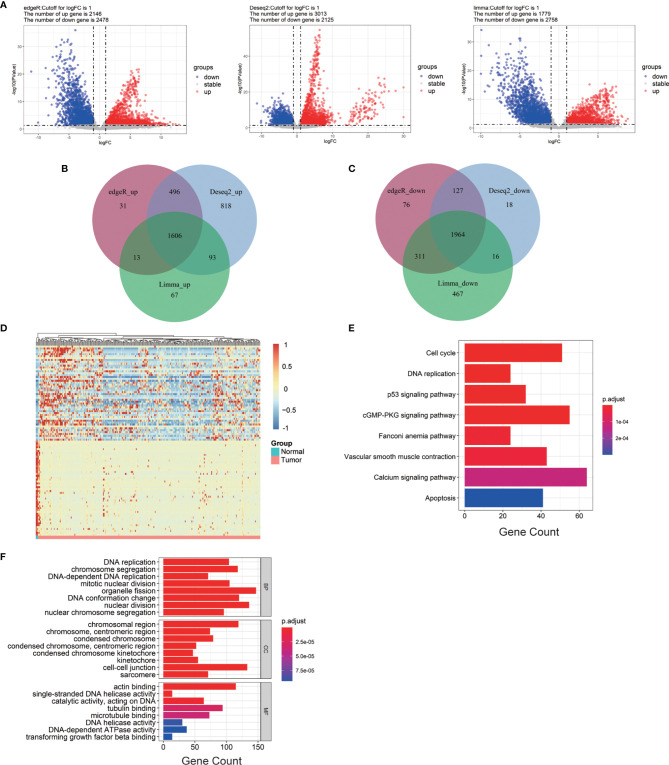
Bioinformatics analysis of all DEGs from cervical cancer tissue samples. **(A)** Volcano plots of DEGs screened by three methods. “Down” refers to the genes that were downregulated. “Up” refers to the genes that were upregulated. “Stable” refers to the genes that have no difference in expression between tumor groups and normal groups. **(B)** Upregulated genes. **(C)** Downregulated genes. **(D)** The heatmap of the top 200 DEGs according to the value of |logFC|. **(E, F)** KEGG pathway enrichment and GO enrichment of DEGs. Each column bar on the y-axis represents an enrichment pathway, and the x-axis is the number of genes that were enriched in this pathway. DEGs, differentially expressed genes; logFC, log fold change; BP, biological process; CC, cellular component; MF, molecular function.

The cervical cancer gene expression profile was collected from TCGA database, including 304 cervical cancer tumor tissues and three normal tissues. EdgeR, DESeq2, and Limma analysis were applied to obtain the DEGs between the normal and cervical cancer groups. The batch information was successively added into the constructed model (George et al., 2014; Nygaard et al., 2016). EdgeR identified 2,146 upregulated genes and 2,478 downregulated genes. DESeq2 identified 3,013 upregulated genes and 2,125 downregulated genes. Limma identified 1,779 upregulated genes and 2,758 downregulated genes (**Figure 1A**). The heatmap of DEGs was generated according to the results from EdgeR, DESeq, and Limma analysis (**Figure S2**). We integrated the results of the three methods to reduce the error, and the results were visualized by Venn diagram (Stupnikov et al., 2021). As shown, 1,606 upregulated and 1,964 downregulated genes were identified (**Figures 1B, C****)**. To narrow down the sample size, we selected 50 highly expressed genes and 50 reduced genes according to the value of |logFC| to generate the heatmap as shown in **Figure 1D**.

To further expand the understanding of the role of all DEGs in the occurrence and progression of cervical cancer, KEGG and GO enrichment analyses were applied. KEGG analysis showed that the selected DEGs were significantly gathered in DNA replication, p53 signaling pathway, and cell cycle (**Figure 1E**). GO enrichment analysis was divided into three groups: BP, CC, and MF. The results of the GO enrichment analysis showed that all of the DEGs in the BP group were enriched in DNA replication, chromosome segregation, and organelle fission. In group CC, these DEGs were highly gathered in cell–cell junction, chromosomal region, actin-binding, single-stranded DNA helicase activity, and catalytic activity were significantly enriched in group MF (**Figure 1F**).

### Weighted Gene Co-Expression Network Analysis Highlighted Seven Key Hub Modules Associated With Clinical Phenotypes of Cervical Cancer

**Figure 2:**
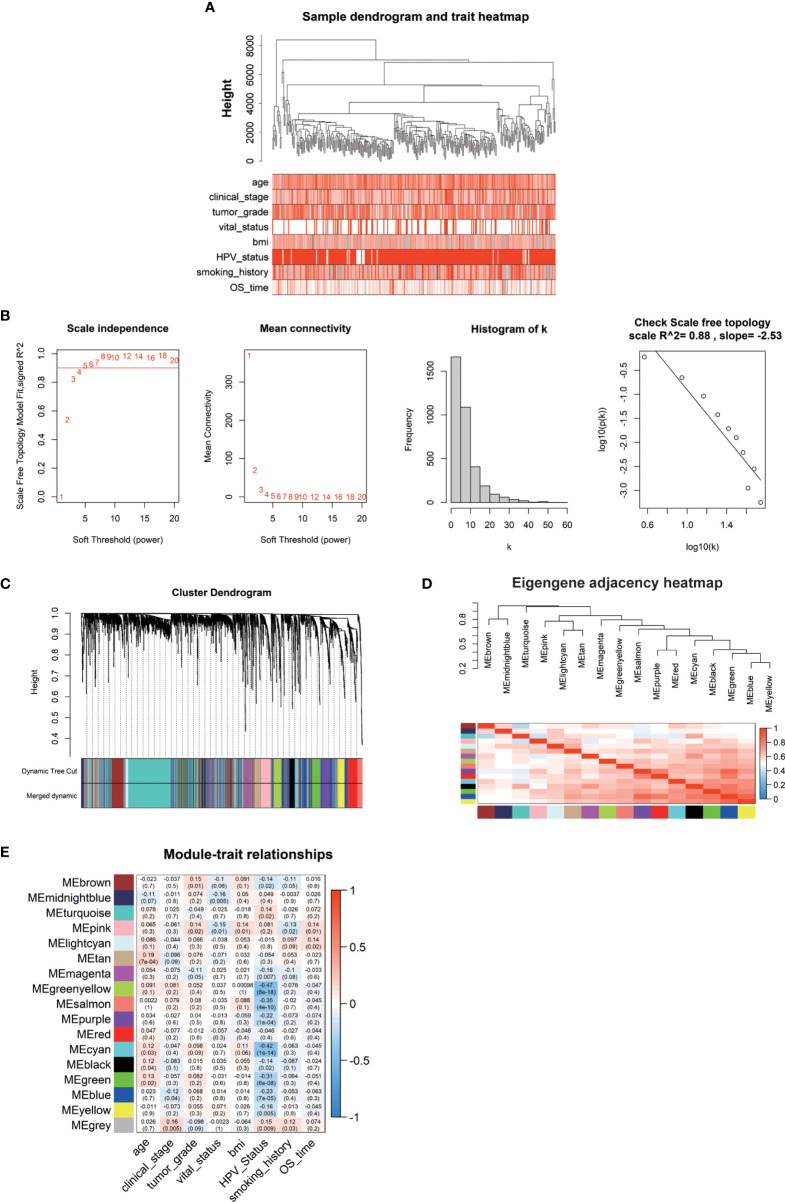
**(A)** The clinical trait heatmap and tree dendrogram. **(B)** Determination of soft-threshold power in the WGCNA. **(C)** Clustering dendrogram of DEGs with assigned module colors. **(D)** Visualization of gene network using heatmap plot. **(E)** Heatmap of the correlation between module eigengenes and phenotype of CESC.

The weighted gene co-expression network was established by the DEGs above methods screened to correlate with clinical phenotypes of cervical cancer. The outlier sample TCGA-LP-A4AV-01A was excluded in subsequent analysis (**Figure S3**). The selected samples were grouped in different clusters to form the distribution map of the clinical feature data, including the age of patients with cervical cancer, clinical stage (stage I–stage IV), histological tumor grade (G1–G3), HPV infection status (negative, positive), body mass index (BMI), patient smoking history, survival status (alive, dead), and survival time (**Figure 2A**). Next, to identify the optimal value of the threshold power from 1 to 30, we conducted network topology analysis to determine the relatively balanced scale independence and mean connectivity of the WGCNA. When the threshold power of β = 4 (scale-free R2 = 0.88) and cutoff modules size, more than 30 were set as the soft threshold to ensure a scale-free network (**Figure 2B**). The tree was grouped into 17 modules by a dynamic tree cut algorithm. All the selected genes were clustered using a TOM-based dissimilarity measurement (**Figure 2C**). Each module was represented by different colors, and the number of genes was concluded and shown in **Table 1**. The genes that did not belong to any of the modules were marked as a gray module. Therefore, this gray module was not included in the subsequent analysis. We analyzed the 16 modules to investigate the interaction between modules, and the heatmap of the network was created (**Figure 2D**). The results indicated that each module was highly independent, but the gene expression in each module was less independent. WGCNA further established the correlation of each module according to different phenotypic traits of cervical cancer by calculating the module significance of correlation of each module-trait (**Figure 2E**). Next, we investigated if each module was positively or negatively correlated, indicating the negatively correlated modules as the following: green-yellow vs. HPV infection status (r = -0.47, p = 8e-18), midnight-blue vs. survival status (r = -0.16, p = 0.005), blue vs. clinical stage (r = -0.12, p = 0.04), and survival time vs. smoking history (r = 0.13, p = 0.02); and the positively correlated modules as the following: tan vs. patient’s age (r = 0.19, p = 7e-04), brown vs. histological grade of cervical cancer (r = 0.15, p = 0.01), pink blue vs. BMI (r = 0.14, p = 0.01), and light cyan vs. survival time (r = 0.14, p = 0.02). Taken together, we selected these seven modules as the most clinical phenotype-associated key hub modules.

**Table 1:** The number of genes in each module.

Module	Genes
Brown	279
midnightblue	41
turquoise	844
Pink	143
lightcyan	40
Tan	77
magenta	140
greenyellow	112
salmon	72
Purple	129
Red	167
Cyan	63
Black	158
Green	179
Blue	716
Yellow	218
grey	192

### Functional Enrichment Analysis Identified 76 Hub Genes From the Hub Modules

**Figure 3:**
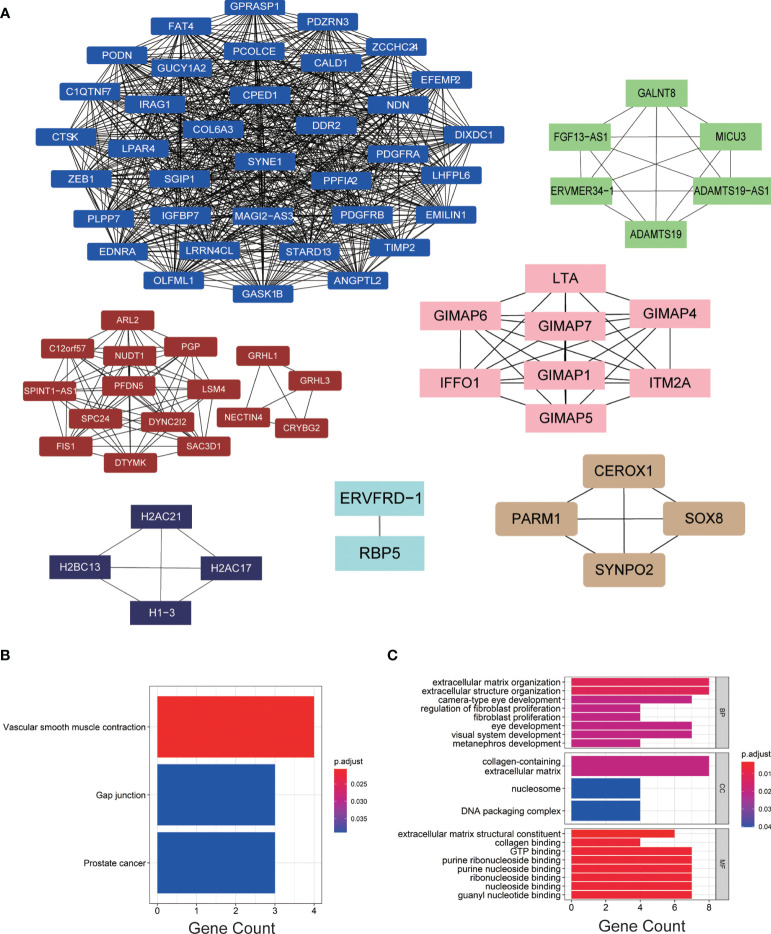
**(A)** The hub genes of each module. **(B, C)** KEGG and GO enrichment analyses of hub genes.

We imported the hub modules into Cytoscape software to screen the hub genes. According to the connectivity between genes in the module, we selected the highest connectivity genes in the modules as hub genes. A total of 76 hub genes were screened from 7 hub modules (**Figure 3A**). The KEGG enrichment analysis indicated that hub genes were gathered in “vascular smooth muscle contraction, gap junction, and prostate cancer” (**Figure 3B**). The GO enrichment analysis showed that “extracellular structure organization, extracellular matrix organization, and camera-type eye development” were significantly gathered in the BP group; “collagen-containing extracellular matrix, nucleosome and DNA packaging complex” were significantly enriched in the CC group; and “collagen binding, GTP binding, and extracellular matrix structural constituent” were significantly enriched in the MF group (**Figure 3C**).

### Five Prognostic Markers for Cervical Cancer Were Highlighted by Cox Regression Analysis

**Figure 4:**
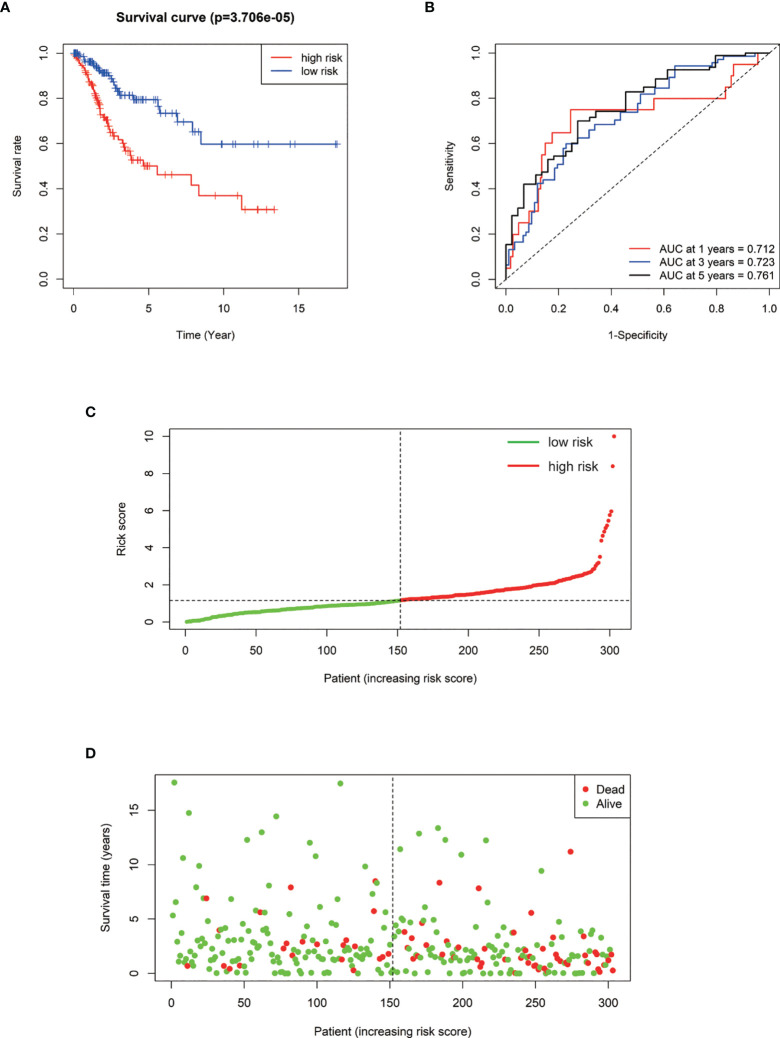
**(A)** Kaplan–Meier survival curve of overall survival between the high‐risk group and low‐risk group. **(B)** The 1-, 3-, 5‐year survival time‐dependent ROC curve. **(C, D)** The distributions of the risk score and survival status for each patient.

We performed a prognostic analysis on the above 76 hub genes to predict the probable prognostic markers. Univariate Cox proportional hazards regression analysis demonstrated that the most significant prognostic factors were EFEMP2, GIMAP4, DYNC2I2, ITM2A, GIMAP7, FGF13-AS1, H1-3, LTA, GIMAP1, and GIMAP5. Multivariate Cox proportional hazards regression analysis was performed to analyze these 10 prognostic factors. GIMAP4, DYNC2I2, EFEMP2, FGF13-AS1, and GIMAP1 showed significant prognostic values, as shown in **Table 2**. Risk values for each patient were obtained from the survival packages. Based on the median risk score, patients with cervical cancer were divided into high-risk and low-risk group. The Kaplan-Meier (KM) survival curve compares the survival time of the high- and low-risk group. As shown, the survival time of the high-risk group was significantly lower than that of the low-risk group (p < 0.0001, **Figure 4A**). To further verify the accuracy of the predicting model of the cervical cancer patient’s survival time, we calculated the area under the curve (AUC) values of Receiver Operating Characteristic Curve (ROC) curves, which is normally adopted to reflect the reliability of the model (AUC >0.7). The AUC value of 1-, 3-, and 5-year survival time prediction model in this study was 0.712, 0.723, and 0.761, respectively, indicating that this model possessed optimal performance in predicting the survival time of cervical cancer patients at 1, 3, and 5 year (**Figure 4B**). We also analyzed the distribution of risk scores and survival status among cervical cancer patients to further confirm the accuracy of our model in predicting cervical cancer patients’ survival time (**Figures 4C, D****)**.

**Table 2:** The significantly prognostic genes revealed by univariate and multivariate Cox proportional hazards regression analysis.

characteristic	Univariate analysis			Multivariate analysis		
	HR	95%CI	P value	HR	95%CI	P value
EFEMP2	1.055	(1.015,1.097)	0.007	1.045	(1.005,1.088)	0.028
GIMAP4	0.941	(0.899,0.984)	0.008	0.829	(0.727,0.945)	0.005
DYNC2I2	0.989	(0.980,0.997)	0.008	0.986	(0.977,0.994	0.001
ITM2A	0.886	(0.808,0.970)	0.009	–	–	–
GIMAP7	0.931	(0.881,0.984)	0.012	–	–	–
FGF13-AS1	8.432	(1.506,47.224)	0.015	13.504	(2.161,84.378)	0.005
H1-3	0.623	(0.423,0.917)	0.016	–	–	–
LTA	0.548	(0.328,0.914)	0.021	–	–	–
GIMAP1	0.652	(0.453,0.939)	0.021	2.545	(0.980,6.614)	0.055
GIMAP5	0.111	(0.017,0.735)	0.023	–	–	–

## Discussion

Cervical cancer is one of the most common tumors among women globally. More than 600,000 new cases are diagnosed by the end of 2020; the death rate of HPV-related cervical cancer in Asia is more than 50%. Low- and middle-income countries have higher mortality rates due to poor medical conditions (Daniyal et al., 2015; Denny, 2015; Vu et al., 2018). The occurrence and progression of cervical cancer are highly associated with the infection of high-risk HPV (Walboomers et al., 1999; Schiffman et al., 2016). In addition, increasing evidence suggested that many DEGs are expressed by cancer cells (van Wieringen and van der Vaart, 2015). Aberrant gene expression levels in the cancer cell may lead to the dysregulation of cell signaling pathways by inhibiting or stimulating (Vogelstein and Kinzler, 2004). Therefore, we detected abnormally expressed genes of CESC from TCGA database. EdgeR, DESeq2, and Limma were applied to reduce errors. A total of 3,570 DEGs were obtained (1,606 were upregulated and 1,964 were downregulated). To further investigate the role of these DEGs in cervical cancer, KEGG analysis and GO enrichment analysis was performed. We observed that these DEGs were significantly enriched in DNA replication, cell cycle, cGMP-PKG signaling pathway, p53 signaling pathway, and Fanconi anemia (FA) pathway. The high-risk E6 oncoprotein has been shown to degrade p53, resulting in the inhibition of apoptosis, which is a key step to progress to cervical cancer (Howie et al., 2009; Kajitani et al., 2012; Zheng et al., 2020). Previous studies demonstrated that the E7 oncoprotein affects cell cycle progression by preventing the binding of the tumor suppressor protein pRB and E2F or degrading pRB and the pocket proteins, which together contribute to cervical cancer progression (Roman and Munger, 2013). Enriched DEGs in the p53 signaling pathway and cell cycle that were highlighted in this study are meaningful to future studies to reveal the mechanism of oncoproteins E6 and E7 in the occurrence of cervical cancer. Plenty of reports have shown that the cGMP/PKG pathway is involved in the proliferation, differentiation, and apoptosis of cancer cells (Fajardo et al., 2014). Furthermore, HPV infection highly depends on cell proliferation and differentiation; a previous study indicated that the cGMP/PKG pathway plays a key role in the malignant phenotype of cervical cancer cells (Gong et al., 2019), and our data are also in agreement with the role of the cGMP/PKG pathway in the development of cervical cancer. The disruption of the FA pathway has been shown to increase HPV16 E7 protein levels and viral genome amplification (Hoskins et al., 2012). Unfortunately, due to the insufficient number of normal samples in TCGA database, there will be a certain amount of errors in screening the DEGs. Even though the DEGs highlighted by our bioinformatics analysis need to be further investigated, the role of these DEGs may provide chances to develop novel drug targets or diagnostic markers.

WGCNA is a co-expression network algorithm, which is widely used in the research of cancer markers. Although a previous study identified many prognostic markers in cervical cancer using WGCNA, they mainly focus on DEGs between groups (tumor vs. normal) (Liu et al., 2019). Still, the clinical phenotype of patients has not been taken into consideration. In addition to HPV infection, previous studies have shown that age, smoking, and obesity are also associated with cervical cancer (Waggoner et al., 2006; Gu et al., 2013; Su et al., 2018; Zhang et al., 2019). Moreover, we also considered that clinical indicators may associate with cervical cancer patients. Therefore, in this study, we firstly screened DEGs between normal tissues and tumor tissues, then carried out WGCNA by considering eight clinical phenotypes of the cervical cancer patient, including the age, clinical stage, tumor histology grade, vital status, BMI, HPV infection status, smoking history, and survival time of cervical cancer patients. Finally, a total of 7 modules were associated with these clinical traits. From the module–trait relationship in WGCNA, HPV infection is the most relevant phenotype. Normally, the HPV infection will be cleared by the host immune system within a year or two. In some rare cases, the HPV may develop into persistent infection, after several years, sometimes decades, may progress to cervical cancer (Hu and Ma, 2018). Interestingly, in our study, WGCNA indicated that older age is positively related to cervical cancer. Considering that about 99.7% of cervical cancer cases are related to high-risk HPV infection, we suspected that older age that positively related to the occurrence of cervical cancer may be due to high-risk HPV persistent infection.

Currently, the prognosis of patients with cervical cancer is not ideal, and the survival rate of patients is too low (Eskander and Tewari, 2014). Therefore, prognostic markers are of great significance to enhance the overall survival rate of patients. To establish a reliable prognostic model, we performed a prognostic analysis of all key genes from the above seven modules. Finally, we identified that the model constructed by GIMAP4, GIMAP1, FGF13-AS1, EFEMP2, and DYNC2I2 could be used as the prediction model for the prognosis of cervical cancer. GIMAP1 and GIMAP4 are proteins of the GIMAP members (GTPase immune-associated proteins) family. GIMAP4 plays a key role in cellular apoptosis and T-cell development, which can be used as a prognostic marker of cervical cancer (Schnell et al., 2006; Heinonen et al., 2015; Xu et al., 2021). GIMAP1 is crucial for the survival of B cells used as the major marker of endometrial cancer (Krucken et al., 1999; Webb et al., 2016; Guo et al., 2021). The human EFEMP2 gene is located near the centromere of chromosome 11q13 and plays a role in the invasion and metastasis of tumors (Obaya et al., 2012; Papke and Yanagisawa, 2014). EFEMP2 has been found related to survival rate; downregulation of EFEMP2 leads to a higher death rate in bladder cancer (Zhou et al., 2019). FGF13-AS1 is a long-chain non-coding RNA (lncRNA); studies have shown that FGF13-AS1 inhibits the proliferation and migration of breast cancer by impairing glycolysis and dry properties. Reduction of FGF13-AS1 is associated with poor prognosis (Ma et al., 2019). DYNC2I2 is involved in cell cycle progression, apoptosis, and gene regulation [provided by RefSeq, March 2014], but this gene was not reported in the literature. Considering the features of DEGs in the expression, clinical phenotypes, and survival time of cervical cancer patients, we concluded that these five hub genes are likely to play a role in cervical cancer, which can be considered as potential biomarkers. However, the function of these five genes needs further experimental verification.

## Data Availability Statement

The data that support the findings of this study are available in The Cancer Genome Atlas (TCGA) at [https://cancergenome.nih.gov/]. These data were derived from the following resources available in the public domain: [https://xenabrowser.net/datapages/?dataset=TCGA-HNSC.htseq_counts.tsv&host=https%3A%2F%2Fgdc.xenahubs.net&removeHub=https%3A%2F%2Fxena.treehouse.gi.ucsc.edu%3A443]. [https://xenabrowser.net/datapages/?dataset=TCGA-HNSC.htseq_fpkm.tsv&host=https%3A%2F%2Fgdc.xenahubs.net&removeHub=https%3A%2F%2Fxena.treehouse.gi.ucsc.edu%3A443].

## Author Contributions

The authors confirm contribution to the paper as follows: Study conception and design: CW; Data collection: HC and TH; Analysis and interpretation of results: HC and RM; Draft article preparation: HC, GX, and CW.All authors reviewed the results and approved the final version of the article.

## Funding

This work was supported by the National Natural Science Foundation of China (grant 82072287).

## Conflict of Interest

The authors declare that the research was conducted in the absence of any commercial or financial relationships that could be construed as a potential conflict of interest.

## Publisher’s Note

All claims expressed in this article are solely those of the authors and do not necessarily represent those of their affiliated organizations, or those of the publisher, the editors and the reviewers. Any product that may be evaluated in this article, or claim that may be made by its manufacturer, is not guaranteed or endorsed by the publisher.
