# Supplementary material for: Bioinformatics Analysis Highlights Five Differentially Expressed Genes as Prognostic Biomarkers of Cervical Cancer and Novel Option for Anticancer Treatment
Source: Front Cell Infect Microbiol. 2022 Jun 17;12:926348. doi: 10.3389/fcimb.2022.926348 (PMC9247199; doi:10.3389/fcimb.2022.926348)

SUPPLEMENT FIGURE LEGENDS

Figure S1. The flow chart of analysis.

Figure S2. EdgeR (a). DESeq2 (b). Limma (c). The heatmap of the top 200 DEGs according to the value of |logFC|. DEGs: differentially expressed genes; logFC: log fold change.

Figure S3. The clustering dendrogram of samples. An outlier sample above the red line was deleted.

Figure s1

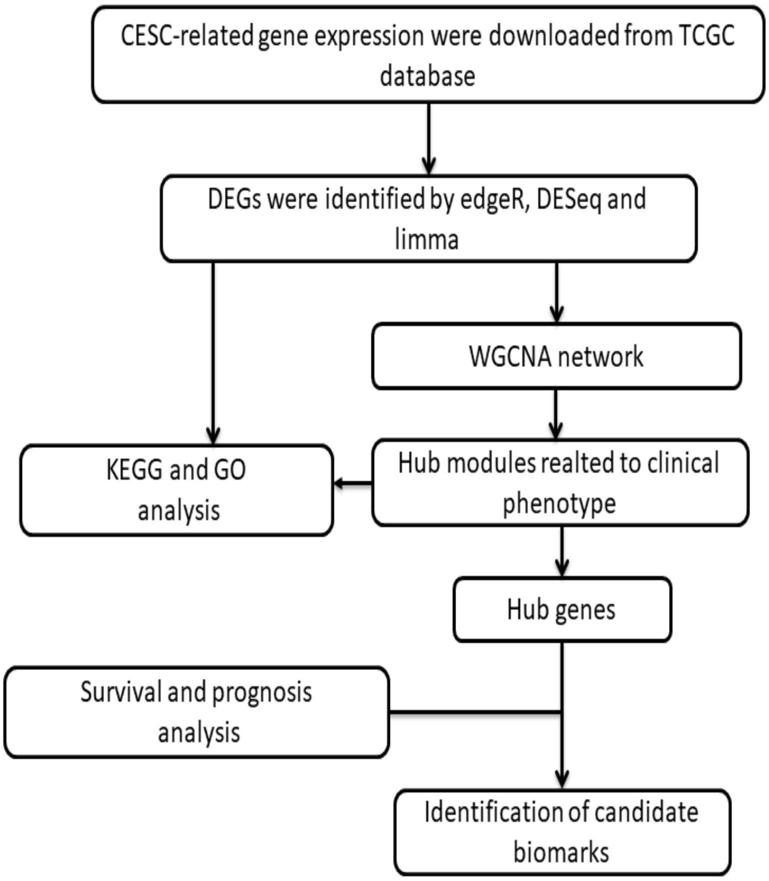

CESC-related gene expression were downloaded from TCGC database
DEGs were identified by edgeR, DESeq and limma
WGCNA network
KEGG and GO analysis
Hub modules realted to clinical phenotype
Hub genes
Survival and prognosis analysis
Identification of candidate biomarks

Figure s2

(a)

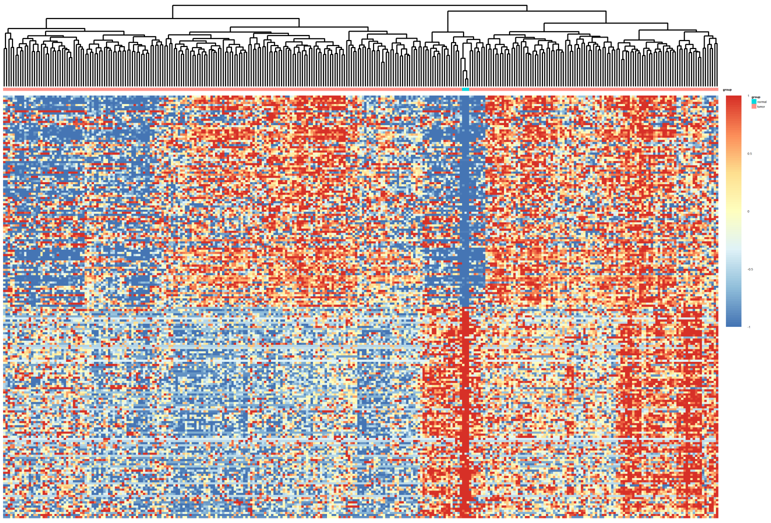

(b)

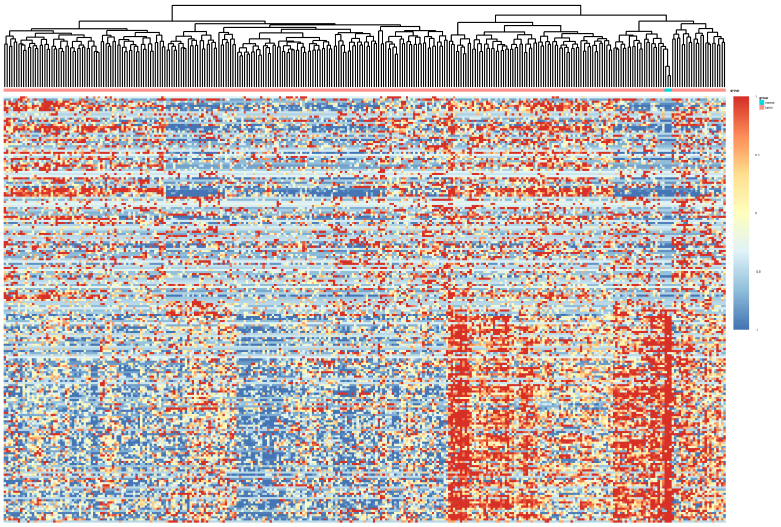

(c)

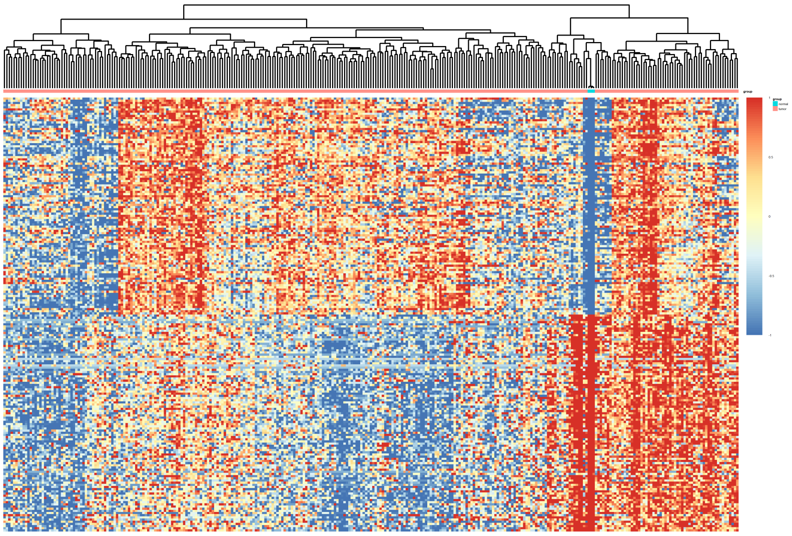

Figure s3

Sample clustering to detect outliers

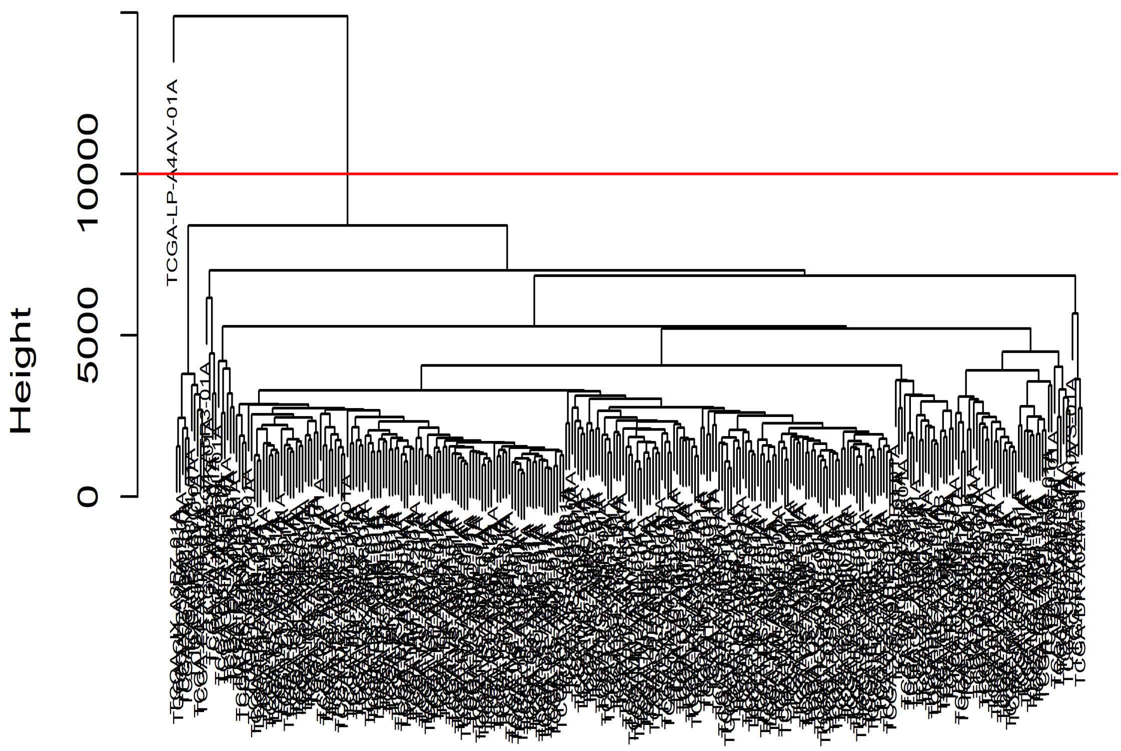

Supplement: Supplementary file 1 [file DataSheet_1.pdf]
